# Effects of High Salt Stress on Secondary Metabolite Production in the Marine-Derived Fungus *Spicaria elegans*

**DOI:** 10.3390/md9040535

**Published:** 2011-03-31

**Authors:** Yi Wang, Zhenyu Lu, Kunlai Sun, Weiming Zhu

**Affiliations:** Key Laboratory of Marine Drugs, Chinese Ministry of Education, School of Medicine and Pharmacy, Ocean University of China, Qingdao 266003, China; E-Mails: wangyi@hotmail.com (Y.W.); zhenyulu1981@yahoo.com.cn (Z.L.); sunqinlai@126.com (K.S.)

**Keywords:** *Spicaria elegans*, high salt stress, secondary metabolites

## Abstract

To obtain structurally novel and bioactive natural compounds from marine-derived microorganisms, the effect of high salt stress on secondary metabolite production in the marine-derived fungal strain, *Spicaria elegans* KLA-03, was investigated. The organism, which was isolated from marine sediment, produced different secondary metabolites when cultured in 3% and 10% saline conditions. Four characteristic metabolites, only produced in the 10% salinity culture, were isolated, and their structures were identified as (2*E*,2′*Z*)-3,3′-(6,6′-dihydroxybiphenyl-3,3′-diyl)diacrylic acid (**1**), aspulvinone E (**2**), aspochalasin E (**3**) and trichodermamide B (**6**), according to their 1D and 2D NMR spectra. Compound **1** is a new compound. High salt stress may therefore be a promising means to induce the production of new and chlorinated compounds in halotolerant fungi. Compound **1** showed moderate antibacterial activity against *Pseudomonas aeruginosa* and *Escherichia coli* with minimum inhibitory concentration (MIC) values of 0.038 and 0.767 mM, respectively.

## 1. Introduction

The marine environment is an important source of halotolerant microorganisms. During our studies of halotolerant “talented strains” [1], we have previously reported that the marine-derived fungal strain, *Spicaria elegans* KLA-03, produces many interesting, bioactive compounds in different media containing 3% salt [2–4]. In the course of our ongoing investigations of structurally new and bioactive compounds from halotolerant fungi in hypersaline media [5–9], and to investigate whether high salt stress could induce *S. elegans* KLA-03 to produce new compounds, the secondary metabolites of *S. elegans* KLA-03, which had been cultivated under conditions of 10% salinity, were studied. As a result, a new metabolite, (2*E*,2′*Z*)-3,3′-(6,6′-dihydroxybiphenyl-3,3′-diyl) diacrylic acid (**1**), together with five known compounds, aspulvinone E (**2**) [10], aspochalasin E (**3**) [11], aspergillazine A (**4**) [2], and trichodermamides A (**5**) [2] and B (**6**) [12] were isolated and identified. Compounds **1**–**3** and **6** were not produced by *S. elegans* KLA-03 when cultivated in a low-salt (3%) medium [2–4].

## 2. Results and Discussion

### 2.1. Identification of Metabolites from *S. Elegans* in 10% Saline Conditions

**Figure 1.**
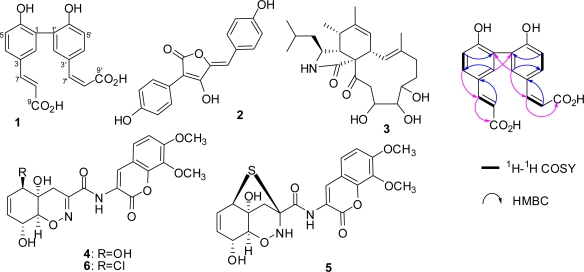
Structures of metabolites **1**–**6** produced by *S. elegans* when cultured in 10% saline conditions, and key COSY and HMBC correlations used to assign the structure of the new compound **1**.

Fungus *S. elegans* KLA-03 was incubated in a high-salt medium containing 10% artificial sea salt and extracted with EtOAc to afford a crude extract. The crude extract (4 g) was separated by extensive chromatography using silica gel, Sephadex LH-20 and HPLC to give compounds **1**–**6** (Figure 1). Compound **1** was isolated as a white powder. Its molecular formula was established as C_18_H_14_O_6_ by HRESIMS at *m/z* 325.0716 [M − H]^−^ (calculated for C_18_H_13_O_6_, 325.0712). The IR spectrum showed the presence of hydroxy groups (3430, 3215 cm^−1^), a conjugated carbonyl group (1677 cm^−1^), double bond (1617 cm^−1^) and benzene ring (1499 cm^−1^). The UV absorption at *λ*_max_ 300 nm indicated that a conjugated system was present in **1**. Two sets of coupled ^1^H NMR signals at δ_H_: 7.64 (dd, *J* = 8.7, 2.3 Hz), 7.57 (d, *J* = 2.3 Hz), 6.87 (d, *J* = 8.7 Hz) and 7.52 (dd, *J* = 8.7, 2.3 Hz), 7.40 (d, *J* = 2.3 Hz), 6.91 (d, *J* = 8.7 Hz) revealed the presence of two 1,3,4-trisubstituted aromatic fragments. Another two sets of coupled proton signals at δ_H_: 7.52 (d, *J* = 15.6 Hz), 6.29 (d, *J* = 15.6 Hz) and 6.80 (d, *J* = 12.8 Hz), 5.74 (d, *J* = 12.8 Hz) suggested the presence of two 1,2-disubstituted double bonds with *E*- and *Z*-configurations, respectively. Four exchangeable proton signals at δ_H_ 12.22 (2H) and 9.82 (2H) were also observed by ^1^H NMR. The DEPT ^13^C NMR spectrum exhibited 18 signals, and indicated the presence of two 3,4-disubstituted phenylpropenoic acid moieties. This assignment was supported by HMBC correlations from H-7 to C-9 and C-4, from H-7′ to C-9′, and C-4′, from H-8 to C-3, and from H-8′ to C-3′ (Table 1). Key HMBC correlations between H-2 and C-1′, and H-2′ and C-1, revealed that the two phenylpropenoic acid moieties were connected via a C_3_−C_3′_ single bond (Figure 1). Thus, compound **1** was determined to be (2*E*,2′*Z*)-3,3′-(6,6′-dihydroxybiphenyl-3,3′-diyl)diacrylic acid.

### 2.2. Effect of Salt Stress on Secondary Metabolite Production

**Figure 2.**
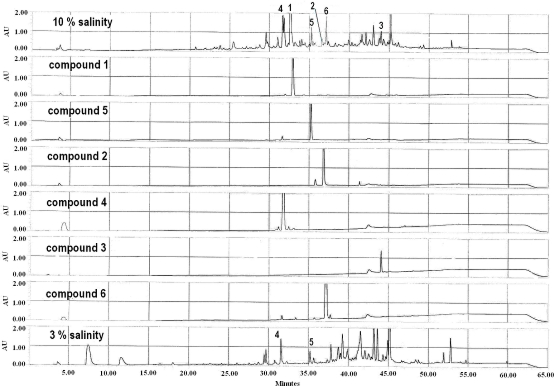
HPLC profiles of secondary metabolites from *S. elegans* KLA-03 at 3% and 10% salinity (gradient elution: 0–5 min, 5% MeOH; 5–50 min, 5%–100% MeOH; 50–60 min, 100% MeOH; 60–65 min, 100%–5% MeOH).

Little has been published in the literature on the metabolites produced by the fungus *S. elegans*, with most reports originating from our lab. A series of compounds, mainly cytochalasins and aspochalasins with antitumor activities, were isolated and identified from the cultivation of this organism at 3% salinity [2–4,13,14]. It has been reported that halotolerant fungi have special osmoregulatory mechanisms to regulate intracellular osmotic potential, which allows them to survive under a variety of saline conditions, including high salt conditions [15]. The secondary metabolites produced by the organism may be regulated by environmental factors such as salinity [16]. To investigate whether high salt stress could activate silent genes encoding secondary metabolites in *S. elegans* KLA-03, the organism was fermented at 3% and 10% salinity and the metabolites produced were monitored by HPLC. When cultured at 10% salinity, *S. elegans* KLA-03 was found to produce different compounds to those observed when it was cultured at 3% salinity (Figure 2).

The main products observed under low salinity culture conditions, cytochalasins (*t*_R_ 37–45 min) [8,9], were present at significantly lower levels when the organism was cultured under high salinity conditions. In contrast, the levels of other metabolites (*t*_R_ 28–35 min) were significantly higher in the high salinity culture. High salinity conditions therefore induced the fungus *S. elegans* KLA-03 to produce much more polar metabolites. Extraction and HPLC-guided purification resulted in the isolation and identification of one new compound (**1**) and five known compounds (**2**–**6**), of which compounds **4** and **5** had previously been identified from the fermentation broth of this strain using 3% salinity conditions [2]. The biosynthetic pathways expressed by *S. elegans* KLA-03 under high salinity conditions are therefore different from those expressed under low salinity conditions. Chlorinated metabolite **6** was not produced at 3% salinity, suggesting that a halogenase enzyme may be activated under high salt conditions, and that the high concentration of chloride was exploited by *S. elegans* KLA-03 to synthesize chlorinated compounds. The mass of the crude EtOAc extracts obtained under the two conditions were markedly different, with 4 g obtained from a 50 L fermentation broth at 10% salinity, while 15 g was obtained from a 15 L 3% saline culture [3,4]. This suggests that high salt conditions lead to significantly lower overall metabolite productivity, as well as a change in metabolite profile.

### 2.3. Antimicrobial Effects of Secondary Metabolites

The antimicrobial activities of compounds **1**–**6** against *Enterobacter aerogenes*, *Escherichia coli*, *Pseudomonas aeruginosa*, *Staphylococcus aureus* and *Candida albicans* were evaluated using an agar dilution method (Table 1) [17]. New compound **1** showed moderate antibacterial activity against *P. aeruginosa* and *E. coli*. In addition, moderate antibacterial activity against *E. aerogenes* for compounds **2**–**6**, and *P. aeruginosa* for **4** and **6** were also observed. The minimum inhibitory concentrations (MICs) were defined as the lowest concentration at which no microbial growth could be observed.

Aspochalasin E (**3**) and trichodermamide B (**6**) have been reported to be cytotoxic to HCT-116 cells, with IC_50_ values of 6.3 and 0.32 μg/mL, respectively [11,12]. Compounds **4** and **5** have been reported to display weak cytotoxicity against HL-60 cells, with IC_50_ values of 84 and 89 μM, respectively [2].

## 3. Experimental Section

### 3.1. General Experimental Procedures

UV spectra were recorded on a Beckman DU 640 spectrophotometer. IR spectra were obtained on a Nicolet NEXUS 470 spectrophotometer as KBr disks. ^1^H, ^13^C and DEPT NMR spectra and 2D-NMR spectra were recorded on a JEOL JNMECP 600 spectrometer using TMS as an internal standard, and chemical shifts were recorded as δ values. ESIMS was measured on a Q-TOF Ultima Global GAA076 LC mass spectrometer. Semipreparative HPLC was performed using an ODS column (YMC-pack ODS-A, 10 × 250 mm, 5 μm, 4 mL/min). Analytical HPLC was performed using an ODS column (YMC-pack C18, 4.6 × 250 mm, 5 μm, 2 mL/min). TLC was performed on plates precoated with silica gel GF_254_ (10–40 μm) and column chromatography (CC) was carried out using silica gel (200–300 mesh, Qingdao Marine Chemical Factory, Qingdao, China) and Sephadex LH-20 (Amersham Biosciences, Sweden). Artificial sea salt used in culture media was purchased from Qingdao Marine Chemical Factory, Qingdao, China.

### 3.2. Fungal Material

The fungus *S. elegans* KLA-03 was isolated from marine sediments collected in Jiaozhou Bay, China. It was identified by Prof. C. X. Fang and preserved in the China Center for Type Culture Collection (No. CCTCCM 205049). Working stocks were prepared on potato dextrose agar slants and stored at 4 °C.

### 3.3. Fermentation and Extraction

The same incubation and extraction methods were used for the 10% artificial sea salt culture as those used previously for culturing with 3% sea salt [8]. In brief, the fungus *S. elegans* KLA-03 was grown under static conditions at 25 °C for 25 days in 180 1 L conical flasks containing a liquid medium (300 mL/flask) composed of glucose (20 g/L), peptone (5 g/L), malt extract (3 g/L), yeast extract (3 g/L), artificial sea salt (10 g/L) and tap water, after adjusting the pH of the media to 7.0. The fermented whole broth (54 L) was filtered through cheesecloth to separate the supernatant from the mycelia. The supernatant was concentrated under reduced pressure to about 5 L and then extracted three times with EtOAc to give an EtOAc extract, while the mycelia were extracted three times with acetone. The acetone was removed under reduced pressure to afford a residual aqueous solution. This aqueous solution was extracted three times with EtOAc to give a further EtOAc crude extract. The EtOAc solutions were then combined and concentrated under reduced pressure to give a final crude extract (4.0 g).

### 3.4. Purification

The extract (4.0 g) from *S. elegans* cultured in 10% saline medium was separated into three fractions on a silica gel column using a step gradient elution of CHCl_3_–MeOH. Fraction 3 was further purified by chromatography on an ODS column, eluted using gradient elution of MeOH–H_2_O, to provide three fractions. The sixth fraction obtained from this purification step (Fraction 3–6) was further separated on a silica gel column to provide compound **4** (15.3 mg), and 13 subfractions. Fractions 3-6-9 and 3-6-13 were initially purified using Sephadex LH-20 (CHCl_3_:MeOH, 1:1) and further purified using semipreparative HPLC to give **1** (3 mg, *t*_R_ 11.50 min, isocratic elution with 55% MeOH) and **5** (5.4 mg, *t*_R_10.38 min, 50% MeOH), respectively. Fraction 3–11 was partially purified by chromatography on Sephadex LH-20 (CHCl_3_:MeOH, 1:1) and further purified on a silica gel column eluted with a gradient of CHCl_3_–MeOH to provide five subfractions. Fraction 3-11-2-1 was further purified by semipreparative HPLC to give compound **3** (14.2 mg, *t*_R_ 11.7 min, isocratic elution with 55% MeOH). Compounds **2** and **6** (2.5 mg, *t*_R_ 16.15 min, and 9.7 mg, *t*_R_ 12.78 min, respectively, isocratic elution with 55% MeOH) were purified from Fraction 3-11-2-2 by semipreparative HPLC.

(2*E*,2′*Z*)-3,3′-(6,6′-Dihydroxybiphenyl-3,3′-diyl)diacrylic acid (**1**): white amorphous powder; UV (MeOH) *λ*_max_ 300 nm; IR (KBr) *ν*_max_ 3430, 3215, 1617, 1499 cm^−1^; ^1^H and ^13^C NMR, see Table 2. HRESIMS *m*/*z* 325.0716 [M − H]^–^ (calcd 325.0712 for C_18_H_13_O_6_).

### 3.5. Bioassays

Antimicrobial activities against *E. aerogenes*, *E. coli*, *P. aeruginosa*, *S. aureus* and *C. albicans* were evaluated using an agar dilution method [17]. The tested strains were cultivated in LB agar plates for bacteria and in YPD agar plates for *C. albicans*, at 37 °C. Compounds **1**–**6** and positive controls were dissolved in 5% DMSO-H_2_O at different concentrations from 1000 to 62.5 μg/mL and then from 50 to 0.78 μg/mL, using continuous 2-fold dilution. The test solutions (5 μL) were absorbed onto paper disks (5 mm diameter) and placed on the assay plates. After 24 h incubation, zones of inhibition (mm in diameter) were recorded. The minimum inhibitory concentrations were defined as the lowest concentration at which an inhibition zone could be observed.

## 4. Conclusions

In summary, four metabolites, including a new compound and a chlorinated compound with antibacterial activity, were identified from the fungus *S. elegans* KLA-03 grown under high salt stress conditions. High salt stress affected the overall quantity of secondary metabolites produced and the metabolite profile. This indicates that the regulation of salinity could be a promising way to obtain new compounds and chlorinated compounds from halotolerant fungi for drug screening.

## Figures and Tables

**Table 1.** Antimicrobial activities of compounds **1**–**6** (“−” indicates not measured).

	**MIC (*m*M)**				
**Compound**	***Enterobacter aerogenes***	***Escherichia coli***	***Pseudomonas aeruginosa***	***Staphylococcus aureus***	***Candida albicans***
**1**	0.153	0.038	0.767	1.534	0.383
**2**	0.084	0.338	3.378	3.378	0.844
**3**	0.059	0.239	1.193	2.387	0.298
**4**	0.029	0.231	0.231	0.231	0.289
**5**	0.056	0.223	2.227	0.223	0.557
**6**	0.056	0.222	0.222	0.222	0.278
Ciprofloxacin lactate	0.001	0.004	0.030	0.001	−
Ketoconazole	−	−	−	−	0.005

**Table 2.** ^1^H- and ^13^C-NMR (600 and 150 MHz) data for **1** in DMSO-*d*_6_.

**position**	***δ*_C_**	***δ*_H_ (*J* in Hz)**	**HMBC (H→C)**	**^1^H-^1^H COSY**
1	126.0 s			
2	131.9 d	7.40 (1H, d, 2.3)	1′, 4, 7	
3	125.0 s			
4	128.6 d	7.52 (1H, dd, 8.7, 2.3)	2, 6, 7	5
5	116.0 d	6.91 (1H, d, 8.7)	1, 3	4
6	157.2 s			
7	144.3 d	7.52 (1H, d, 15.6)	2, 4, 9	8
8	115.3 d	6.29 (1H, d, 15.6)	3, 9	7
9	167.9 s			
1′	124.8 s			
2′	134.2 d	7.57 (1H, d, 2.3)	1, 4′, 7′	
3′	125.5 s			
4′	131.1 d	7.64 (1H, dd, 8.7, 2.3)	2′, 6′, 7′	5′
5′	115.1 d	6.87 (1H, d, 8.7)	1′, 3′	4′
6′	156.0 s			
7′	141.5 d	6.80 (1H, d, 12.8)	2′, 4′, 9′	8′
8′	117.0 d	5.74 (1H, d, 12.8)	3′, 9′	7′
9′	167.7 s			
